# Phosphorus Necrosis

**Published:** 1890-05-03

**Authors:** 


### PHOSPHORUS NECROSIS.

Since the almost universal substitution of the less active red for the white phosphorus in the manufacture of matches, the horrible destruction of the bones of the jaws and nose produced by the inhalation of the fumes has happily become a rare occurrence. Still, without strict precautions, the handling of even the red form is not free from danger, and cases of necrosis of the jaws present themselves from time to time at hospitals in the vicinity of large match factories. Dr. H. Haeckel, of Jena, has published in detail the history, phenomena, and treatment of fifty-six cases coming under his personal observation. A careful study of these has convinced him that the only hope of cure is to be found in the early and complete extirpation of every trace of diseased bone, and that resections not extending beyond the alveolar processes are useless. Thus, when necrosis has attacked the region of the incisors, or the canine teeth, the middle portion of the lower jaw must be excised; if that of the bicuspids or molars be affected, then the entire half of the jaw on that side should be removed; and when both sides are involved, the whole jaw must be sacrificed. In such cases it is better to excise the two rami at separate sittings, taking the worst side first, and allowing two or even three months to elapse between the operations, though in urgent cases the entire jaw may be cut out at one time. If, however, the pain is comparatively unimportant, the suppuration moderate, and the general health little impaired, it is well to wait long enough to permit of the subosteal method of procedure. In disease of the upper jaw it is no less necessary to insist on thoroughness, and to deprecate any partial resection, promising as such less radical operations may appear soon after performance. Nor should the surgeon be misled by cases of apparently spontaneous healing in parts with separation of small sequestra, for here the danger to life, consequent on the extension of the disease, is much greater, its insidious progress occasionally revealing itself only by the supervention of basal meningitis, while the deformity, inconvenience, and functional disturbance caused by removal of large portions of the superior maxilla are far less than those attending excision or resection of the lower.

Other cases of necrosis of the jaw not due to phosphorus poisoning are almost always traceable to a carious tooth as their starting point. But though the existence of a specific, idiopathic, or rheumatic form of the disease, maintained by some surgeons, has long been doubted or denied by the majority of surgeons, Dr. Herzog describes a case in which, while all extraneous causes could be excluded, the entire half jaw from chin to joint was more or less involved, and the suppuration so prolonged that complete separation of the periosteum took place over nearly half the anterior, and in patches on the posterior surface of the bone. Dr. Weil, a dental surgeon at Berlin, has seen in all four such cases, each following unusual exposure to cold and wet.

